# Impact of PD-L1 Scores and Changes on Clinical Outcome in Rectal Cancer Patients Undergoing Neoadjuvant Chemoradiotherapy

**DOI:** 10.3390/jcm9092775

**Published:** 2020-08-27

**Authors:** Florian Huemer, Eckhard Klieser, Daniel Neureiter, Verena Schlintl, Gabriel Rinnerthaler, Franck Pagès, Amos Kirilovsky, Carine El Sissy, Wolfgang Iglseder, Franz Singhartinger, Tarkan Jäger, Adam Dinnewitzer, Nadja Zaborsky, Markus Steiner, Richard Greil, Lukas Weiss

**Affiliations:** 1 Department of Internal Medicine III with Haematology, Medical Oncology, Haemostaseology, Infectiology and Rheumatology, Oncologic Center, Salzburg Cancer Research Institute-Laboratory for Immunological and Molecular Cancer Research (SCRI-LIMCR), Paracelsus Medical University, 5020 Salzburg, Austria; f.huemer@salk.at (F.H.); v.schlintl@salk.at (V.S.); g.rinnerthaler@salk.at (G.R.); n.zaborsky@salk.at (N.Z.); mark.steiner@salk.at (M.S.); r.greil@salk.at (R.G.); 2 Institute of Pathology, Paracelsus Medical University Salzburg, 5020 Salzburg, Austria; e.klieser@salk.at (E.K.); d.neureiter@salk.at (D.N.); 3 Cancer Cluster Salzburg, 5020 Salzburg, Austria; 4 Laboratory of Integrative Cancer Immunology, INSERM UMRS1138, Immunology and Cancer Department, Cordeliers Research Center, 75006 Paris, France; franck.pages@aphp.fr (F.P.); amos.kirilovsky@gmail.com (A.K.); carineelsissy@hotmail.com (C.E.S.); 5 Hôpital Européen Georges Pompidou, Assistance Publique-Hôpitaux de Paris, Université de Paris, Faculté de santé, 75015 Paris, France; 6 Department of Radiation Oncology, Paracelsus Medical University Salzburg, 5020 Salzburg, Austria; w.iglseder@salk.at; 7 Department of Surgery, Paracelsus Medical University Salzburg, 5020 Salzburg, Austria; f.singhartinger@salk.at (F.S.); ta.jaeger@salk.at (T.J.); adam.dinnewitzer@ooeg.at (A.D.)

**Keywords:** programmed death-ligand 1, TPS, CPS, IC, survival

## Abstract

Reports on the prognostic role of programmed death-ligand 1 (PD-L1) expression in rectal cancer are controversial. We investigated expression patterns and changes of PD-L1 in rectal cancer patients undergoing neoadjuvant chemoradiotherapy (CRT). Seventy-two patients diagnosed with rectal cancer and/or treated with fluorouracil-based neoadjuvant CRT at the Department of Internal Medicine III of the Paracelsus Medical University Salzburg (Austria) between January 2003 and October 2012 were included. PD-L1 scoring was performed according to the tumor proportion score (TPS), combined positive score (CPS), and immune cell score (IC). PD-L1 TPS prior to neoadjuvant CRT had a statistically significant impact on survival (median: ≤1%: 95.4 months (95% CI: 51.8—not reached) vs. >1%: not reached, *p* = 0.03, log-rank). Patients with a PD-L1 TPS ≤1% prior to and after CRT showed an inferior survival compared to all other patients (median: 56.7 months (95% CI: 51.4—not reached) vs. not reached, *p* = 0.005, log-rank). In multivariate analysis, PD-L1 TPS prior to neoadjuvant CRT (>1% vs. ≤1%, hazard ratio: 0.29 (95% CI: 0.11–0.76), *p* = 0.01) remained independently associated with survival. In conclusion, low PD-L1 TPS was associated with inferior survival in rectal cancer patients undergoing neoadjuvant CRT. A prospective validation of the prognostic value of PD-L1 expression in rectal cancer patients within a clinical trial is necessitated.

## 1. Introduction

The treatment of stage II-III rectal cancer usually consists of neoadjuvant capecitabine or 5-fluorouracil (5-FU)-based long-course chemoradiotherapy (CRT) followed by surgery [1,2]. Despite the application of adjuvant chemotherapy, 32–43% of rectal cancer patients experience a disease relapse within the first six years [3]. Immune checkpoints, such as programmed death-ligand 1 (PD-L1), can be therapeutically targeted, which has led to dramatic clinical improvements in various tumor entities [4,5,6,7,8,9,10,11,12]. However, in metastatic colorectal cancer (CRC), the clinical benefit of this therapeutic approach is almost exclusively restricted to tumors with microsatellite instability (MSI) [13,14,15] and the addition of an immune-checkpoint inhibitor to adjuvant chemotherapy in stage III colon cancer with MSI is currently being investigated in the ATOMIC trial [16] (NCT02912559). Studies specifically investigating the prognostic role of PD-L1 expression in rectal cancer are sparse. The applied anti-PD-L1 monoclonal antibodies, the time point of PD-L1 testing, as well as the applied PD-L1 scores were heterogeneous in these studies and the impact of PD-L1 expression on clinical outcome was controversial [17,18,19,20,21]. The aim of our retrospective single-center analysis was to evaluate PD-L1 expression patterns and time-dependent changes according to three established PD-L1 scores and the impact on clinical outcome in a well-defined rectal cancer cohort undergoing neoadjuvant CRT.

## 2. Experimental Section

This research project was approved by the local ethics committee of the Provincial Government of Salzburg, Austria (415-EP/73/655-2016).

### 2.1. Patients

Patients with TNM stage I–IV rectal cancer diagnosed and/or treated at the tertiary cancer center of the Paracelsus Medical University Salzburg (Austria) with neoadjuvant capecitabine/5-FU-based CRT followed by total mesorectal excision were consecutively selected for this retrospective analysis. Inclusion requirements were the availability of the paraffin-embedded diagnostic primary tumor sample as well as of the primary tumor specimen obtained from definitive surgery. Decisions on the initiation of neoadjuvant CRT and follow-up care were based on the respective National Comprehensive Cancer Network (NCCN) and European Society of Medical Oncology (ESMO) treatment guidelines for rectal cancer. Radiotherapy consisted of 45.0 Gy of radiation delivered to the primary tumor, the mesorectal lymph nodes, pre-sacral lymph nodes, and internal iliac lymph nodes. The chemotherapy backbone was either oral capecitabine or infusional 5-FU as monochemotherapy or in combination with oxaliplatin. Surgery was performed six to eight weeks after completion of neoadjuvant CRT. Adjuvant chemotherapy was recommended based on interdisciplinary tumor board decisions on an individual basis. Patients were classified as low (<8), intermediate (8–16), or high (>16) risk according to the neoadjuvant rectal (NAR) score [22], which incorporates the pathological nodal stage and primary tumor downstaging during neoadjuvant CRT.

### 2.2. Immunohistochemistry

Immunohistochemical examination was performed on 4-µm formalin-fixed paraffin-embedded (FFPE) sections. Each case was raised on adhesive glass slides and dried at 60 °C for one hour. Deparaffination, antigen retrieval, immunostaining, counter staining, dehydration, and cover slip application as well as pre-treatment were conducted using standardized routine immunohistochemistry (IHC) protocols. Immunohistochemical staining was performed either on a Ventana Benchmark Ultra instrument (Ventana Medical Systems, Tucson, AZ, USA; trademark of Hoffmann-La Roche AG, Basel, Switzerland) or on a Dako Omnis Autostainer combined with the EnVision Plus System (Dako, Vienna, Austria). The anti-PD-L1 ready-to-use antibody (22C3, SK006, Agilent, Santa Clara, CA, USA) was used for PD-L1 staining. Each sample was assessed by two experienced pathologists according to the tumor proportion score (TPS: (PD-L1-stained tumor cells/total number of viable tumor cells) × 100) [23], combined positive score (CPS: (PD-L1-stained tumor cells and immune cells/total number of viable tumor cells) × 100) [24], and immune cell score (IC: (PD-L1-stained immune cells/total number of viable tumor cells) × 100) [25] in diagnostic biopsies prior to neoadjuvant CRT as well as in specimens obtained from definitive surgery after neoadjuvant CRT. The PD-L1 assessors were blinded to clinical outcome. Sections were incubated with anti-CD3 (2GV6, Ventana Medical Systems, Tucson, AZ, USA; trademark of Hoffmann-La Roche AG, Basel, Switzerland; Oro Valley, United States) and anti-CD8 (SP57 Ventana Medical Systems, Tucson, AZ, USA; trademark of Hoffmann-La Roche AG, Basel, Switzerland; Oro Valley, United States) ready-to-use primary antibodies to assess CD3^+^ as well as CD8^+^ T cell density (cells/mm^2^) in diagnostic biopsies prior to neoadjuvant CRT.

### 2.3. Microsatellite Status

MSI testing (Idylla^TM^ MSI Test, Biocartis, Mechelen, Belgium) was performed according to the manufacturer’s recommendations. In short, one or more 10-µm FFPE sections containing ≥20% neoplastic cells and an overall tissue area of >25 mm^2^ were used per analysis. The sections were sandwiched in nuclease-free water wetted Whatman filter papers (grade 1, 10 mm circles, GE healthcare, Buckinghamshire, GB) and placed inside the MSI cartridge and measured with the Idylla^TM^ instrument. Seven MSI biomarkers were simultaneously analyzed (ACVR2A, BTBD7, DIDO1, MRE11, RYR3, SEC31A, SULF2). The analysis was considered valid if ≥5 out of these 7 biomarkers showed valid marker results. A sample was acknowledged as “microsatellite instability-high” (MSI-H) if ≥2 biomarkers were found to be mutated. Otherwise, the sample was classified as “microsatellite stable” (MSS).

### 2.4. Tumor Regression Grade

The tumor regression grade (TRG) after neoadjuvant CRT was assessed by the Dworak regression score based on the ratio of viable tumor cells to fibrosis (ranging from 0 to 4); TRG 4 defined a pathologic complete remission (pCR) [26]. Patients achieving a TRG 3 or TRG 4 were classified as responders to neoadjuvant CRT, and patients with a TRG 0–2 were classified as non-responders.

### 2.5. Statistical Analysis

For PD-L1, established dichotomized cut-off values from phase III clinical trials [5,11,12,27] as well as quartiles were chosen to assess the impact on clinical outcome. Kaplan–Meier survival curves together with log-rank testing were used to compare survival distributions between patient groups. Disease-free survival (DFS) was calculated from the date of surgery of the primary tumor until the date of relapse or date of last known follow-up for stage I–III rectal cancer patients. Patients without recurrence at the last contact were censored. Overall survival (OS) was calculated from the date of initial diagnosis of rectal cancer stage I–III until the date of death or date of last known follow-up. Patients alive at the last contact were censored. Stage IV rectal cancer patients were excluded from DFS and OS analyses. Continuous data, such as age, were summarized using medians and ranges and compared between groups with the Mann–Whitney test. Correlations were tested using the Spearman test. Parameters that proved statistically significant in univariate analysis (*p* < 0.05) were included in multivariate analysis. *p*-values < 0.05 were considered to indicate statistical significance. SPSS IBM (version 23.0, New York, USA) and R (version 3.5.1, www.R-project.org, Vienna, Austria) including “package” survival were used for statistical analysis.

## 3. Results

### 3.1. Patient and Treatment Characteristics

This retrospective analysis was based on the data of 72 rectal cancer patients diagnosed and/or treated at the tertiary cancer center in Salzburg, Austria, between January 2003 and October 2012. The baseline characteristics are depicted in Table 1.

### 3.2. PD-L1 Expression Prior to Neoadjuvant CRT and PD-L1 Changes after Completion of CRT

**Figure 1:**
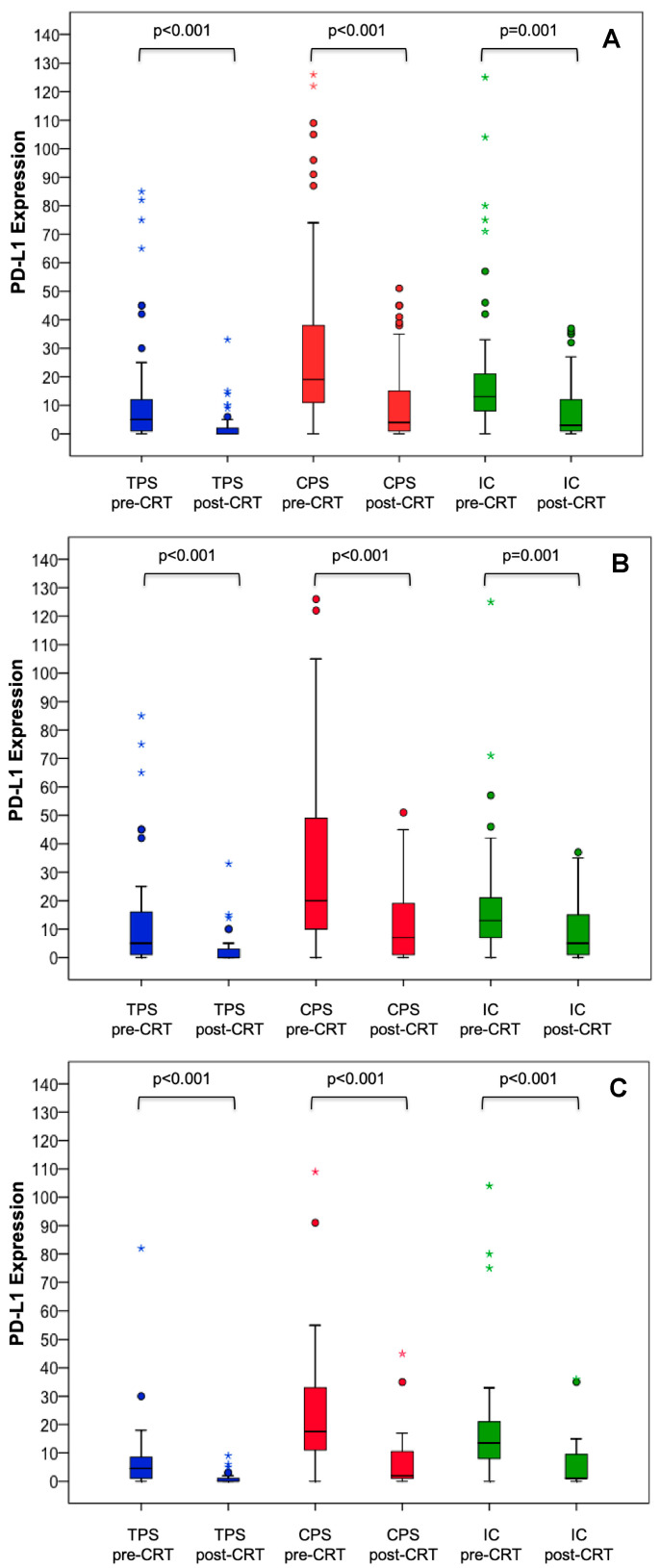
PD-L1 TPS, CPS, and IC changes during neoadjuvant CRT. PD-L1 TPS, CPS and IC changes during neoadjuvant CRT (**A**) in the entire cohort, (**B**) in patients with doublet chemotherapy, and (**C**) in patients with monochemotherapy. CPS: combined positive score; CRT: chemoradiotherapy; IC: immune cell score; PD-L1: programmed death-ligand 1; TPS: tumor proportion score.

PD-L1 expression status prior to neoadjuvant CRT was evaluable in 70 (97%) patients. PD-L1 positivity (≥1%) according to TPS, CPS, and IC was found in 93%, 97%, and 97%, respectively. PD-L1 expression significantly decreased after completion of neoadjuvant CRT according to TPS (median: 4.0 vs. 0.0, *p* < 0.001), CPS (median: 18.5 vs. 3.0, *p* < 0.001) and IC (median: 13.0 vs. 3.0, *p* = 0.001) (Figure 1A). The decline of PD-L1 expression was independent of the chemotherapy backbone (5-FU/oxaliplatin-based doublet chemotherapy vs. 5-FU-based monotherapy, Figure 1B,C). PD-L1 expression and CD8^+^ T cell density prior to neoadjuvant CRT showed a statistically significant positive correlation across all PD-L1 scores (TPS: *r* = 0.28, *p* = 0.03; CPS: *r* = 0.32, *p* = 0.01; IC: *r* = 0.28, *p* = 0.03). A statistically significant positive correlation between PD-L1 expression and CD3^+^ T cell density was found for IC (*r* = 0.30, *p* = 0.02); this was not the case for TPS (*r* = 0.10, *p* = 0.45) or CPS (*r* = 0.24, *p* = 0.06).

### 3.3. PD-L1 Expression Prior to Neoadjuvant CRT and Association with Surrogate Endpoints

**Figure 2:**
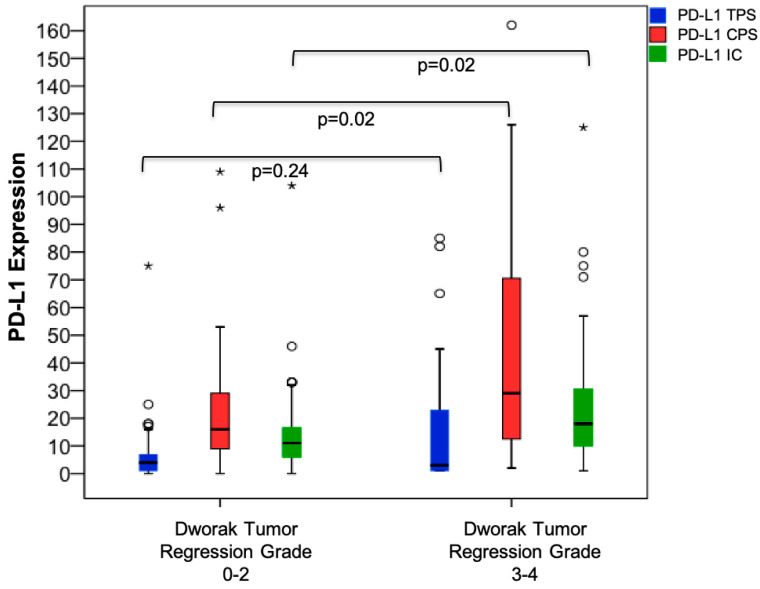
Association between PD-L1 expression prior to neoadjuvant CRT and Dworak tumor regression grade. CPS: combined positive score; CRT: chemoradiotherapy; IC: immune cell score; PD-L1: programmed death-ligand 1; TPS: tumor proportion score. * Outliers above quartile 3 (Q3) + 1.5× interquartile range. * Outliers above quartile 3 (Q3) + 3× interquartile range.

Tumor regression was statistically significantly associated with PD-L1 CPS (Dworak TRG 0–2: 16.0 (median) vs. Dworak TRG 3–4: 29.0 (median), *p* = 0.02) and PD-L1 IC (Dworak TRG 0–2: 11.0 (median) vs. Dworak TRG 3–4: 18.0 (median), *p* = 0.02) prior to neoadjuvant CRT, while this was not the case for PD-L1 TPS (Dworak TRG 0–2: 4.0 (median) vs. Dworak TRG 3–4: 3.0 (median), *p* = 0.24) (Figure 2). The pCR rate did not correlate with PD-L1 TPS (pCR: 11.0 vs. non-pCR: 3.5, *p* = 0.35), CPS (pCR: 29.0 vs. non-pCR: 17.5, *p* = 0.62), or IC (pCR: 17.5 vs. 13.0, *p* = 0.71) prior to neoadjuvant CRT. PD-L1 expression prior to neoadjuvant CRT did not show an association with the NAR score (TPS: *r* = −0.08, *p* = 0.52; CPS: *r* = −0.13, *p* = 0.29; IC: *r* = −0.11, *p* = 0.37).

### 3.4. PD-L1 Expression and Clinical Outcome

#### 3.4.1. PD-L1 Expression Prior to Neoadjuvant CRT

**Figure 3:**
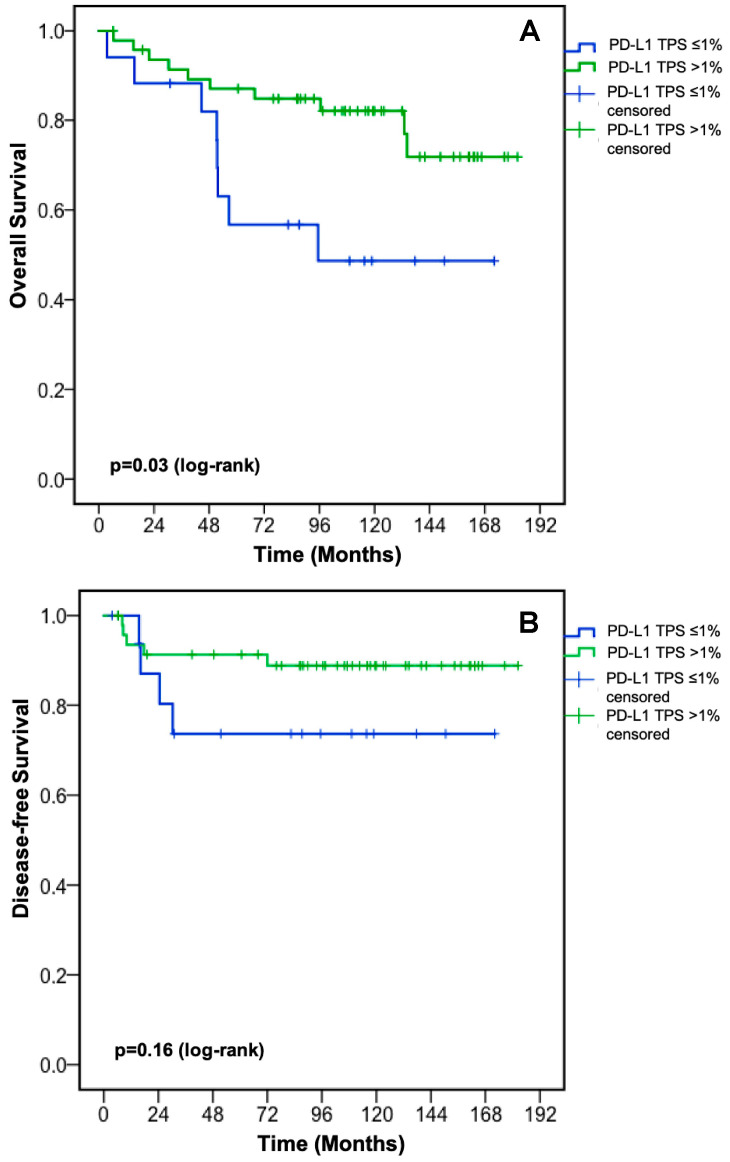
Overall survival (**A**) and disease-free survival (**B**) according to PD-L1 TPS prior to neoadjuvant CRT. CRT: chemoradiotherapy; PD-L1: programmed death-ligand 1; TPS: tumor proportion score. The tick marks on the curves represent censored patients.

Established cut-off values for PD-L1 TPS, CPS, and IC prior to neoadjuvant CRT did not separate rectal cancer patients with different risk profiles for OS (Table A1). By using quartiles (Q) for PD-L1 TPS (Q1: ≤1% vs. Q2–Q4: >1%), a statistically significant survival advantage for rectal cancer patients with higher PD-L1 TPS prior to neoadjuvant CRT was shown (median OS: not reached vs. 95.4 months (95% CI: 51.8—not reached), *p* = 0.03, Figure 3A). Higher PD-L1 TPS (≤1% vs. >1%) was associated with a trend towards better DFS (*p* = 0.16, Figure 3B).

Patient baseline and tumor characteristics according to the PD-L1 TPS status (≤1% vs. >1%) prior to neoadjuvant CRT are depicted in Table A2.

#### 3.4.2. PD-L1 Changes after Completion of CRT

**Figure 4:**
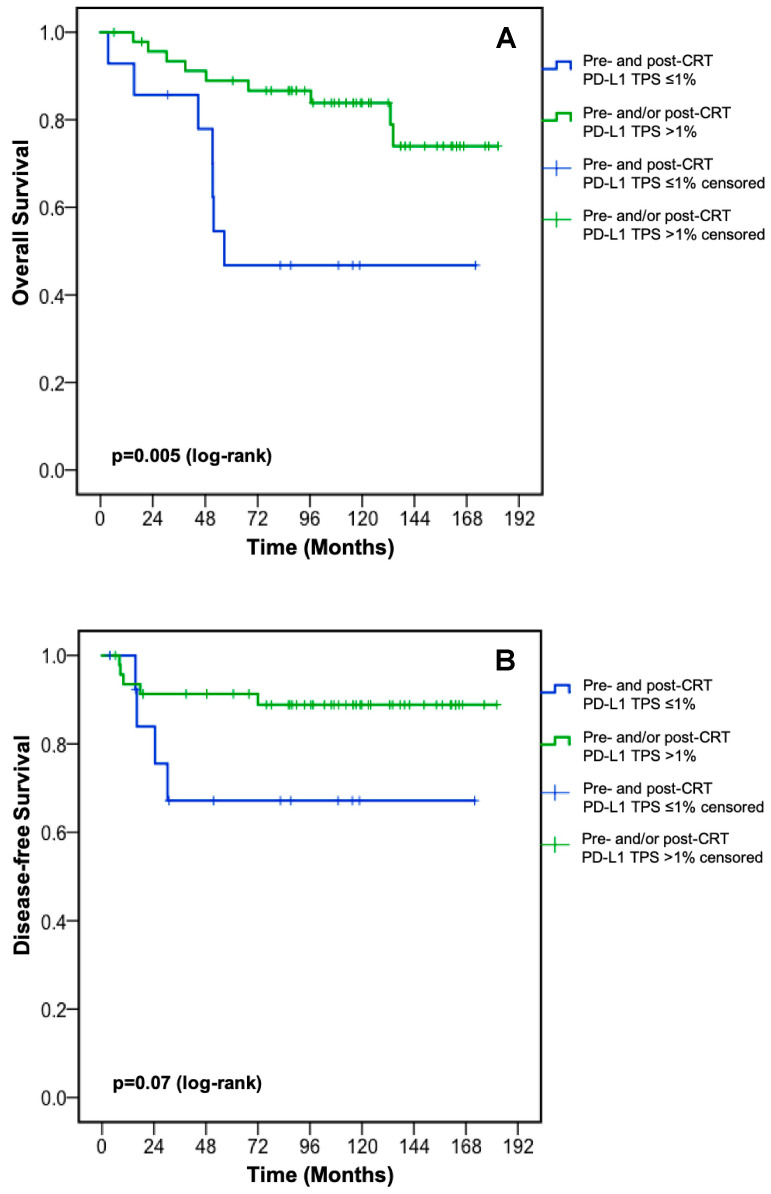
Overall survival (A) and disease-free survival (B) according to PD-L1 TPS changes during neoadjuvant CRT. CRT: chemoradiotherapy; PD-L1: programmed death-ligand 1; TPS: tumor proportion score. The tick marks on the curves represent censored patients.

In order to evaluate the impact of PD-L1 changes on clinical outcome, patients with low PD-L1 TPS (≤1%) prior to and after neoadjuvant CRT were compared to all other patients. Patients with a low PD-L1 TPS at both time points displayed a significantly inferior OS (median OS: 56.7 months (95% CI: 51.4—not reached) vs. not reached, *p* = 0.005, Figure 4A) and a trend towards worse DFS (*p* = 0.07, Figure 4B).

### 3.5. Univariate and Multivariate Analysis for DFS and OS

Sex (male vs. female), age (>65 vs. ≤65 years), ypN stage (positive vs. negative), Dworak TRG (3–4 vs. 0–2), histologic grade (3 vs. 1–2), application of adjuvant chemotherapy (yes vs. no), and PD-L1 TPS prior to neoadjuvant CRT (>1% vs. ≤1%) were tested in univariate analysis and multivariate analysis for DFS and OS. None of these parameters was statistically significantly associated with DFS in univariate analysis (Table 2). Age (>65 vs. ≤65 years, hazard ratio: 3.12 (95%CI: 1.25–7.82), *p* = 0.02) and PD-L1 TPS prior to neoadjuvant CRT (>1% vs. ≤1%, hazard ratio: 0.36 (95%CI: 0.14–0.91, *p* = 0.03) were associated with OS in univariate analysis and remained independently associated with OS in multivariate analysis (age: hazard ratio: 4.09 (95%CI: 1.54–10.86), *p* = 0.005; PD-L1 TPS prior to neoadjuvant CRT: hazard ratio: 0.29 (95%CI: 0.11–0.76), *p* = 0.01) (Table 3). DFS strongly correlated with OS among patients with stage I–III rectal cancer (*r*: 0.91, *p* < 0.001).

## 4. Discussion

By investigating three established PD-L1 scores in this retrospective analysis, we found a statistically significant survival benefit for rectal cancer patients with a higher PD-L1 TPS (>1%) compared to patients with a PD-L1 TPS ≤1% prior to neoadjuvant CRT (Figure 3A). The pre-CRT PD-L1 TPS was not associated with baseline patient and tumor characteristics (Table A2). Other clinically established cut-off values for PD-L1 expression were not associated with OS (Table A1). Our findings of a positive prognostic value of higher PD-L1 TPS prior to neoadjuvant CRT are in line with the publication by Chen et al. [21], while Chiang et al. [19], Hecht et al. [18], and Ogura et al. [20] did not show a statistically significant association between PD-L1 TPS prior to neoadjuvant CRT and OS. The prognostic value of PD-L1 TPS was not restricted to the initial diagnostic biopsy prior to neoadjuvant CRT in our rectal cancer cohort as patients with a PD-L1 TPS ≤1% pre- and post-CRT displayed worse OS compared to patients with PD-L1 upregulation at any time point (Figure 4A). The latter finding suggests that PD-L1 upregulation on cancer cells either before or after CRT is sufficient to improve OS. PD-L1 TPS [19,21] as well as PD-L1 CPS at the invasive front [18] after neoadjuvant CRT were associated with OS in several retrospective analyses. Neoadjuvant chemoradiotherapy is able to trigger interferon-gamma release from cancer cells and in turn to induce PD-L1 expression [19,28]. PD-L1 expression statistically significantly decreased during CRT according to each PD-L1 score in our cohort (Figure 1A); this was independent of the chemotherapy backbone (Figure 1B,C). Hecht et al. also reported a statistically significantly decrease of PD-L1 expression on immune cells after neoadjuvant CRT, whereas no changes of PD-L1 expression on cancer cells were found [18]. An increase of the proportion of PD-L1-positive/high samples (defined by an arbitrary threshold of 5%) during neoadjuvant CRT was described by Chiang et al. (PD-L1 expression on cancer cells) [19], Ogura et al. (PD-L1 expression on immune cells but not on cancer cells) [20], and by Chen et al. (PD-L1 expression on cancer cells) [21]; however, changes of the PD-L1 scores were not reported. Given the known heterogeneity of PD-L1 expression within tumors [29], we cannot exclude bias resulting from the analysis of the initial diagnostic biopsy, and comparison with PD-L1 expression in the surgical resectate, since it only represents a small portion of the tumor.

As an immunosuppressive checkpoint on the one hand and a favorable prognostic factor in rectal cancer undergoing neoadjuvant CRT on the other hand, the role of PD-L1 expression seems contradictory. A putative explanation for this observation is the development of an immunosuppressive tumor microenvironment by PD-L1 upregulation in immunogenic tumors during the course of disease as it has been shown for CRC with MSI [30]. In other words, PD-L1 expression via upregulation of interferon-gamma exhibits the capability of a viable adaptive immune response. Surgical removal of the tumor after neoadjuvant CRT may reinvigorate pre-existing antitumor immune responses and may in turn lead to improved clinical outcome. In a meta-analysis including stage I–IV colon and rectal cancer patients, PD-L1 TPS proved to be a negative prognostic factor for DFS and OS. However, it is noteworthy that PD-L1 expression was statistically significantly associated with right-sidedness and poor differentiation, which are well-known tumor characteristics with a negative prognostic value [31].

Testing PD-L1 TPS (≤1% vs. >1%) prior to neoadjuvant CRT and established risk factors in univariate and multivariate analysis revealed an independent favorable impact of higher PD-L1 TPS on OS (hazard ratio: 0.29, Table 3). Hecht et al. found an independent association between PD-L1 CPS prior to as well as after neoadjuvant CRT and OS [18] and Chiang et al. described an independent impact of PD-L1 TPS after neoadjuvant CRT on survival [19] in multivariate analyses, respectively.

Yoshino et al. reported a 30% pCR rate by incorporating five cycles of the immune-checkpoint inhibitor nivolumab to neoadjuvant CRT. Among patients with a PD-L1 TPS ≥1% prior to neoadjuvant CRT, the pCR rate was doubled [32]. The latter findings are hypothesis generating and suggest therapeutic approaches to induce or increase PD-L1 expression before the application of immune-checkpoint inhibitors. However, the addition of oxaliplatin to a 5-FU-based CRT (Figure 1B) had no impact on PD-L1 expression changes when compared to a 5-FU-based CRT (Figure 1C). Therapeutic approaches to induce major histocompatibility complex I expression on cancer cells by MEK inhibitors in order to improve immune-checkpoint blockade efficacy have not resulted in better clinical outcome in metastatic CRC [33]. Antiangiogenic therapy in combination with immune-checkpoint blockade promotes the formation of high endothelial venules, thereby increasing tumor-infiltrating lymphocytes in tumor mouse models [34]. In the ongoing “TARZAN“ trial (NCT04017455), the latter therapeutic approach is being investigated by combining nivolumab with bevacizumab during neoadjuvant therapy of localized rectal cancer.

Our retrospective analysis has several limitations. Surrogate end points, such as TRG as well as the NAR score, did not impact OS in our rectal cancer cohort, which was likely attributable to the limited sample size. However, the strong correlation between DFS and OS (*r*: 0.91) corroborates that disease relapse was the predominant cause of death. The addition of oxaliplatin to 5-FU-based neoadjuvant CRT increases pCR rates [35,36] but comes at the cost of a higher frequency of grade 3 and 4 adverse events [37]. More than half of our patients received a 5-FU-based neoadjuvant CRT in combination with oxaliplatin, a therapy combination that is not recommended by the current ESMO [2] or NCCN [1] rectal cancer guidelines. Decisions on an individual patient basis to initiate neoadjuvant CRT with 5-FU and oxaliplatin were based on improved pCR rates and/or DFS rates reported in the CAO/ARO/AIO-04 trial [35,38] and FOWARC trial [36]. However, CRT with 5-FU monotherapy is the standard neoadjuvant therapy protocol at our institution. The inclusion period of rectal cancer patients was 10 years (2003–2012) in this retrospective analysis; however, this time interval was comparable to other reports [18,19,20,21]. The localization of CD3^+^ and CD8^+^ T cells (intratumoral vs. invasive margin) was not taken into account. However, the standardized and validated “immunoscore” in its original version is neither applicable to localized rectal cancer prior to neoadjuvant CRT, nor after CRT due to post-radiogenic alterations. A recently published modified version predicting response to neoadjuvant CRT in localized rectal cancer, the “biopsy-adapted immunoscore”, is solely based on CD3^+^ and CD8^+^ T cell density in the tumor region [39].

In conclusion, PD-L1 TPS prior to neoadjuvant CRT as well as PD-L1 TPS changes showed a statistically significant impact on survival in rectal cancer patients undergoing neoadjuvant CRT, and this was not the case for PD-L1 CPS or PD-L1 IC. PD-L1 TPS prior to neoadjuvant CRT proved as an independent prognostic parameter for OS in multivariate analysis. A clinically relevant OS disadvantage was shown for patients with PD-L1 TPS ≤1% in the initial diagnostic tumor biopsy and in the surgical resectate after neoadjuvant CRT. Considering the published encouraging pCR rates by CRT combined with nivolumab in PD-L1 TPS-positive rectal cancer patients, a sequential therapeutic approach with CRT aiming at upregulating PD-L1 followed by the application of immune-checkpoint blockade might be interesting for initially PD-L1 TPS-negative tumors. The identification of PD-L1-inducing or -upregulating agents for the combination with immune-checkpoint inhibitors in neoadjuvant treatment concepts of rectal cancer will be of utmost importance in future clinical trials. However, a prospective validation of the prognostic value of PD-L1 expression in rectal cancer patients undergoing neoadjuvant CRT is necessitated.

## Figures and Tables

**Table 1:** Baseline characteristics.

Parameter	*N* = 72 (%)
**Age**	
≤65 years	44 (61)
>65 years	28 (39)
**Sex**	
female	23 (32)
male	49 (68)
**ypN stage**	
*N*-	48 (67)
*N*+	24 (33)
**cTNM stage**	
I	1 (1)
II	29 (40)
III	37 (52)
IV	5 (7)
**Histologic grade**	
I	1 (1)
II	61 (87)
III	8 (12)
NA	2
**Dworak tumor regression grade**	
0	3 (4)
I	16 (22)
II	25 (35)
III	20 (28)
IV	8 (11)
**Microsatellite status**	
MSS	56 (98)
MSI	1 (2)
NA	15
**CRT backbone**	
5-FU or capecitabine	29 (40)
5-FU + oxaliplatin or capecitabine + oxaliplatin	43 (60)
**NAR score**	
low	11 (15)
intermediate	36 (50)
high	25 (35)
**Adjuvant chemotherapy**	
yes	36 (52)
no	33 (48)
NA	3

CRT: chemoradiotherapy; MSI: microsatellite instability; MSS: microsatellite stability; NA: not available, NAR score: neoadjuvant rectal score; PD-L1: programmed death-ligand 1.

**Table 2:** Univariate and multivariate analysis for disease-free survival

	UVA		MVA	
	HR (95% CI)	*p*-Value	HR (95% CI)	*p*-Value
**Sex** male (*n* = 45) female (*n* = 22)	1.86 (0.39–8.95)	0.44	−	−
**Age** >65 (*n* = 25) ≤65 (*n* = 42)	0.56 (0.12–2.69)	0.47	−	−
**ypN stage** N+ (*n* = 20) N− (*n* = 47)	1.21 (0.30–4.84)	0.79	−	−
**Dworak TRG** 3–4 (*n* = 28) 0–2 (*n* = 39)	0.74 (0.18–2.95)	0.67	−	−
**Histologic grade** 3 (*n* = 7) 1 + 2 (*n* = 58)	0.20 (0.002–17.98)	0.49	−	−
**Adjuvant chemotherapy** yes (*n* = 35) no (*n* = 30)	0.88 (0.22–3.52)	0.86	−	−
**PD-L1 TPS pre-CRT** >1% (*n* = 48) ≤1% (*n* = 17)	0.40 (0.11–1.50)	0.18	−	−
**NAR score** intermediate/high (*n* = 56) low (*n* = 11)	27.20 (0.02–43,658.20)	0.38	−	−

95% CI: 95% confidence interval; CRT: chemoradiotherapy; HR: hazard ratio; MVA: multivariate analysis; NAR score: neoadjuvant rectal score; PD-L1: programmed death-ligand 1; TPS: tumor proportion score; TRG: tumor regression grade; UVA: univariate analysis.

**Table 3:** Univariate and multivariate analysis for overall survival

	UVA		MVA	
	HR (95% CI)	*p*-Value	HR (95% CI)	*p*-Value
**Sex** male (*n* = 45) female (*n* = 22)	2.24 (0.74–6.76)	0.15	-	-
**Age** >65 (*n* = 25) ≤65 (*n* = 42)	3.12 (1.25–7.82)	0.02	4.09 (1.54–10.86)	0.005
**ypN stage** N+ (*n* = 20) N− (*n* = 47)	1.11 (0.42–2.91)	0.84	-	-
**Dworak TRG** 3–4 (*n* = 28) 0–2 (*n* = 39)	1.10 (0.44–2.75)	0.84	-	-
**Histologic grade** 3 (*n* = 7) 1 + 2 (*n* = 58)	0.67 (0.25–1.84)	0.44	-	-
**Adjuvant chemotherapy** yes (*n* = 35) no (*n* = 30)	0.72 (0.28–1.83)	0.49	-	-
**PD-L1 TPS pre-CRT** >1% (*n* = 48) ≤1% (*n* = 17)	0.36 (0.14–0.91)	0.03	0.29 (0.11–0.76)	0.01
**NAR score** intermediate/high (*n* = 56) low (*n* = 11)	1.89 (0.44–8.17)	0.40	−	−

95% CI: 95% confidence interval; CRT: chemoradiotherapy; HR: hazard ratio; MVA: multivariate analysis; NAR score: neoadjuvant rectal score; PD-L1: programmed death-ligand 1; TPS: tumor proportion score; TRG: tumor regression grade; UVA: univariate analysis.

## Appendix A

**Table A1:** DFS and OS according to established PD-L1 cut-off values.

	DFS		OS	
PD-L1 Expression Prior to CRT (*n*)	Median (months)	*p*-Value (log-rank)	Median (months)	*p*-Value (log-rank)
**TPS**				
<1% (4) ≥1% (61)	24.5 NR	0.04	56.7 NR	0.21
≤5% (39) >5% (26)	NR NR	0.83	NR NR	0.16
≤10% (48) >10% (17)	NR NR	0.27	NR NR	0.11
≤20% (56) >20% (9)	NR NR	0.20	NR NR	0.27
≤50% (61) >50% (4)	NR NR	0.41	NR NR	0.23
**CPS**				
<1 (2) ≥1 (63)	24.5 NR	0.17	44.7 NR	0.32
≤10 (15) >10 (50)	NR NR	0.34	NR NR	0.58
≤20 (34) >20 (31)	NR NR	0.85	134.2 NR	0.05
≤50 (52) >50 (13)	NR NR	0.97	NR NR	0.24
**IC**				
<1% (2) ≥1% (63)	24.5 NR	0.17	44.7 NR	0.32
≤5% (11) >5% (54)	NR NR	0.60	132.9 NR	0.47
≤10% (23) >10% (42)	NR NR	0.87	NR NR	0.66
≤20% (46) >20% (19)	NR NR	0.64	NR NR	0.18
≤50% (59) >50% (6)	NR NR	0.88	NR NR	0.43

CPS: combined positive score; CRT: chemoradiotherapy; DFS: disease-free survival; IC: immune cell score; NR: not reached; OS: overall survival; PD-L1: programmed death-ligand 1; TPS: tumor proportion score.

**Table A2:** Baseline patient and tumor characteristics according to PD-L1 TPS status prior to neoadjuvant CRT.

Parameter	PD-L1 TPS ≤1% *N* = 17 (%)	PD-L1 TPS >1% *N* = 48 (%)	*p*-Value
**Age**			0.755
≤65 years	11 (65)	29 (60)	
>65 years	6 (35)	19 (40)	
**Sex**			0.368
female	4 (23)	17 (35)	
male	13 (77)	31 (65)	
**cTNM stage**			0.559
I	0 (0)	1 (2)	
II	9 (53)	19 (40)	
III	8 (47)	28 (58)	
**cT stage**			0.662
T2	1 (6)	1 (2)	
T3	14 (82)	43 (90)	
T4	2 (12)	4 (8)	
**cN stage**			0.422
N-	9 (53)	20 (42)	
N+	8 (47)	28 (58)	
**Histologic grade**			0.230
I	1 (6)	0 (0)	
II	14 (82)	42 (91)	
III	2 (12)	4 (9)	
NA	0	2	
**Lymphovascular invasion**			0.657
no	11 (85)	30 (79)	
yes	2 (15)	8 (21)	
NA	4	10	
**Venous invasion**			0.069
no	10 (83)	38 (97)	
yes	2 (17)	1 (3)	
NA	5	9	

PD-L1: programmed death-ligand 1; TPS: tumor proportion score.
